# Enhanced independence and quality of life through treatment with flotation-Restricted Environmental Stimulation Technique of a patient with both Attention Deficit Hyperactivity Disorder and Aspergers Syndrome: a case report

**DOI:** 10.4076/1757-1626-2-6979

**Published:** 2009-07-07

**Authors:** Hanna Edebol, Anette Kjellgren, Sven-Åke Bood, Torsten Norlander

**Affiliations:** Department of Psychology, Karlstad UniversitySE-651 88, KarlstadSweden

## Abstract

**Introduction:**

The objective of this qualitative case report was to describe experiences of flotation-Restricted Environmental Stimulation Technique from the perspective of a woman with Attention Deficit Hyperactivity Disorder, Aspergers syndrome and experiences of depression and distress.

**Case presentation:**

The respondent is a 36-year-old woman from Sweden, assessed and diagnosed by a neuropsychological multi-professional team in 2006. The 19-session flotation series prolonged during almost one year.

**Conclusion:**

The positive development of arousal control, activity regulation, sensory integration and interpretation, cognitive functioning and emotional maturity created experiences of personal independence and quality of life. Flotation-restrictive environmental stimulation technique was experienced as a meaningful treatment. Additional studies of treatment for Attention Deficit Hyperactivity Disorder and comorbid disorders in adults using the flotation-restrictive environmental stimulation technique are strongly encouraged.

## Introduction

The neuropsychological disorder Attention Deficit Hyperactivity Disorder (ADHD) includes symptoms like hyperactivity, inattention, impulsivity and distractibility [1]. Another neuropsychological disorder, Aspergers syndrome (AS) is often comorbid with ADHD and include symptoms like repetitive behaviour, atypical thresholds for sensory impressions, complications with communication and social behaviour. Treatment of adults with neuropsychological disorders is often complicated by the great extent of comorbid disorders. Documentation of pharmacotherapy with stimulants demonstrates an average response-rate of 80% [2]. The efficacy of alternative treatments lack consensus, a meta-study of treatments like essential fatty acid supplementation, laser acupuncture, EEG biofeedback, mirror feedback and channel-specific perceptual training concluded that these methods did not entail sufficient amounts of controlled documentation and were not yet studied thoroughly enough to outline any recommendations of treatment [3]. However, the meta-study points at one common theme of many of the alternative treatments; the training of the arousal level [3]. Neurological patterns of arousal and relaxation might be of critical value to understand treatment of neuropsychological disorders.

A well-documented technique of relaxation, flotation-Restricted Environmental Stimulation Technique (flotation-REST), has actively facilitated relaxation in a variety of cases of severe and long-lasting states of stress [4,5]. Flotation-REST is a mild form of sensory isolation and enables a person to float and relax in water due to high levels of saline. The skin-tempered water of the flotation tank minimizes tactile sensations, the insulated lid of the tank allows isolation from sensory stimulation and together these means facilitate the experience of relaxation.

The objective of this study was to carry out an initial qualitative evaluation of flotation-REST based upon experiences of an adult person with ADHD and comorbidity of Aspergers syndrome for the first time in the literature.

## Case presentation

### The respondent

The respondent is a 36-year-old woman from Sweden, diagnosed by a neuropsychological team with ADHD combined type (DSM-IV; 314.01) and Aspergers syndrome (DSM-IV; 299.80) according to the criterions of the DSM-IV [6]. The respondent floated 45 minutes for the first six sessions and for 90 minutes the remaining 13 sessions, a total of 19 sessions. Concerta® (54 mg), which is a stimulant that improves attention and reduces the level of impulsive behaviour in patients with ADHD, was used at the onset of the flotation but not after three months. The antidepressant Efexor® depot (75 mg) was taken daily throughout the flotation treatment.

### The interviews

The respondent contacted the department of psychology with enquiries about the flotation-REST and was included at the waiting list. After she had floated twelve sessions she was asked to describe her experiences from the flotation in a letter and later she agreed to be interviewed about her experiences. She got the information that everything would be treated confidentially. In order to address the reliability, the respondent was informed that some of the quotations would be red by two assessors during a credibility test, as well as included in the presentation of the study later on. The respondent was also informed that she had the right to terminate the interview at any time without giving a motive and without it affecting the treatment.

The respondent was interviewed a total of three times for about an hour each time. The semi-structured interview was used. Examples of questions; *how do you experience ADHD; how do you experience flotation?* During the third interview no interview guide was used.

### The analysis

The Empirical Phenomenological Psychological (EPP) method according to Karlsson [7] was used to analyse the interviews. The EPP-method includes five steps of processing the material; it is read over in order to develop a first picture of the total material; it is divided into meaning units (MU) based upon the underlying psychological meaning of the text; every meaning unit is transformed from spoken language into abstract language in order fully recognize the underlying psychological phenomenon of the material; the material is arranged into clusters of situational structures and finally; the material is arranged into clusters of typological structures which is presented in the results and discussion section.

To control the reliability of the results, the Norlander Credibility Test [8,9], designed for phenomenological analysis, was used. Two assessors were given the task to independently assign 50 transformed MUs into ten of the categories. The correspondence of the assessors were 72 % and 86 % respectively, yielding a mean NCT value of 79 % which is in line with previously published results [8,9]. In order to examine the validity of the typological structures, the respondent was presented with the result and given time to reflect upon them. According to the respondent, the result presents an accurate picture of her experiences.

## Discussion

**Figure 1.**
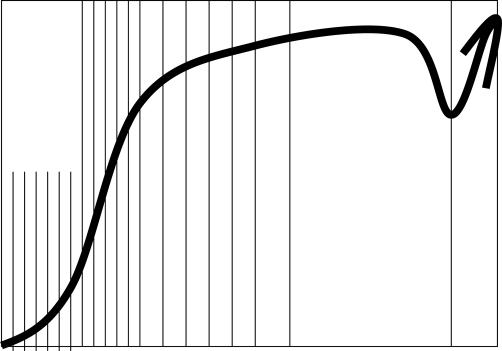
The positive development of the 19-sessions flotation series.

The objective of this qualitative case-study was to gain insight into how an adult person with ADHD, comorbidity of Aspergers syndrome as well as experiences of depression and distress experience relaxation during flotation-REST. The analysis resulted in 31 typological structures which have been arranged into five general themes. The general themes described in these results and discussion section altogether embodies the experience of personal independence and quality of life (Figure 1).

Abilities of *Arousal control* develop as the respondent slowly adjust and achieve adequate levels of arousal by the means of flotation. The respondents’ level of arousal is altered and adjusted during flotation and enables experiences of deep-working relaxation. The flotation enables better abilities of arousal control, deep relaxation and decreased stress. *“I fell asleep there and that is somewhat…the whole point, as to speak, to really gain…true, true relaxation… so that is floating to me, to really…to really be able to relax and wind down”*.

Abilities of *Activity regulation* including the ability to inhibit, control and calibrate physical movements and impulsive activities develop during flotation. Awareness and understanding of the body and its movement patterns as well as control of these functions are included in these abilities. “*I do not have the same restlessness in the body like before, so it is easier for me to sit still”.*

Abilities of *Sensory integration* and *interpretation* develop as reduced levels of stimuli enable the senses to relax and regain resistance. Tolerance and management of impressions are facilitated. The sensory deprivation during flotation also induces experiences of integrated sensory functioning and sensory wholeness. *“It is easier to handle these impressions and filter them out somehow and that is probably because of this inner calm”*.

*Cognitive* abilities concerning concentration, focusation, motivation, preparation and organization improve during the series of flotation. It becomes easier to complete thoughts, plan sequences, focus carefully, concentrate and think methodical. The cognitive abilities develop later in the flotation process and are perceived as deep-rooted. *“It is easier for me to be able to concentrate and that is for example when I am going to read things, when I am concentrating to write things, when I am concentrating in general”*.

*Emotional* abilities of managing and modulating emotions develop during flotation. The emotional abilities lead to experiences of patience and persistence. Personal emotions become easier to accept and handle while other people’s emotions become more relatable. Social and emotional development creates experiences of inner harmony and acceptance. *“Somehow I can accept myself more the way I am and that is probably what comes out of this inner calm that I have been having from the floating, I have gained this inner calm so that I can accept myself the way I am.”*

## Development of personal independence and quality of life

The broad spectra of the abilities create experiences of personal independence which helps the respondent to experience quality of life. In order to maintain independence, support-sessions need to be performed. The abilities sustain during the weekly break from flotation but at the end of a break for several weeks, the personal independence slowly decreases. In this case, one support session was performed two and a half month after the previous session and regained development and independence for several weeks. Figure 1 schematically shows the positive development of the personal independence.

Arousal, activity and sensory abilities develop at an early onset while deep-rooted cognitive and emotional abilities develop later on. This work brought about reflections of how arousal, activity and sensory abilities might precede development of cognitive and emotional abilities. The interrelated aspects of the abilities could be an interesting area for future studies.

Timing of support-sessions are critical, in this case, the break remained for two and a half month, which might have been to long. Even though development reappeared after the support-session, the experience of decreased wellbeing and reduced development at the end of the break indicate that this support-session could have been favourable at an earlier point of time. Positive effects of flotation-REST, like increased energy, optimism and decreased levels of stress remained in a four month follow-up study of patients with stress-related pain [10].

## Conclusion

The objective of this qualitative case study was to gain insight into experiences of flotation-REST from the perspective of a person with ADHD, Aspergers syndrome and experiences of stress and depression. During a series of 19 sessions, positive development of abilities related to arousal control, activity regulation, sensory integration and interpretation, cognitive functioning and emotional maturity created experiences of personal independence. Additional studies of treatment of ADHD and comorbid psychiatric disorders in adults using the flotation-REST are strongly encouraged.

## Abbreviations

ADHD: Attention Deficit Hyperactivity Disorder
AS: Aspergers syndrome
Flotation-REST: Flotation-Restricted Environmental Stimulation Technique
DSM-IV: Diagnostic and Statistical Manual of mental disorders-IV
EPP: Empirical Phenomenological Psychological method
MU: Meaning Unit
NCT: Norlander Credibility Test
